# Topics of Public Interest

**Published:** 1916-08

**Authors:** 


### TOPICS OF PUBLIC INTEREST.

---

**Statistics as to Expense of Sickness.** R. M. Hardy in Public Health, is quoted as saying that in 1915, the American people lost a million years in sickness—a tenth of a year per capita—which is probably a conservative estimate; that

$1,220,000,000 was paid for the doctor bill alone; and that 3/4 of the loss was preventable. As there are somewhat near 122,000 physicians in the country in actual practice—and note that we make no accurate estimate,—this means an average professional income of $10,000. We are inclined to think that this item has been exaggerated 5-10 times. Possibly, in the sanitary and hygienic millenium, 3/4 of the sickness might be avoided but more accurate statistics of certain details show that the increased liability to disease in the senile period of those who have been saved from tuberculosis, typhoid, etc., in early life, or the inevitable adult diseases of the large number saved from infantile death, tend to compensate. While the average age of human beings in civilized countries at death, is very much longer than it was a quarter of a century ago, the actual longevity of those who escape untimely death has not been materially increased and probably never will be. Certainly, under existing conditions, even granting the application of measures shown to be efficient in preventing disease and death, it can not be said that 3/4 of disease is preventable. 1/4 would be a more reasonable estimate. ''Statistics'' of this sort do more harm than good.

---

**Ink Pad as Hygrometer.** Have you noticed that, with the disuse of heating and the presence of considerable degrees of humidity in the outdoor air, your ink pad absorbs more moisture than usual, owing to the glycerine present, and makes the impressions from a rubbed stamp, illegible from excess if ink, that requires blotting?

---

''**Better Doctoring for Less Money**'' by Dr. Richard C. Cabot of Boston, published in the American Magazine for April, with an illustration of the author and his family, has been condemned by the St. Louis Medical Society as a ''gross violation of ethics'' while it is mentioned with approval in the newspaper advertisements of Pierce's Institute of Buffalo. We have previously considered publications of this general nature, and have expressed the opinion that there should be one rule of ethics for all physicians, irrespective of prominence and some means for controlling the publication of articles in lay periodicals. Dr. Cabot's article contains much that deserves professional attention but obviously, what may do much good and be strictly true in a meeting of physicians is very liable to do great harm and to give false impressions when read by laymen as a criticism of the profession by a distinguished member. The members of a congregation assembled for worship, properly designate themselves as sinners, according to divine standards but it would be neither wise nor truthful for a prominent member of the congregation to publish a letter in a newspaper denouncing his fellows in similar terms, to be accepted by critical outsiders according to worldly standards.

---

**Second Class Postage.** Mr. Randall of California has introduced in the House of Representatives, a bill changing the rates for publications as follows: 1st, 2d and 3d zones (within 300 miles) 1 cent a pound; 4th zone (to 1000 miles) 3 cents; 6th zone (to 1400 miles) 4 cents; 7th zone (to 1800 miles) 5 cents; 8th zone (over 1800 miles) 6 cents. This requires some additional trouble in sorting mail but seems reasonable. Periodical literature can no longer be considered as, in the aggregate, distinctly educational, nor requiring special favors at the expense of the P. O. Dept.

---

**N. Y. State Commission for Boy Training.** The Slater-Welch law provides that all pupils in public and private schools, over the age of 8, shall have physical training for not less than 20 minutes daily. The commission includes the Major-General commanding the National Guard, a member appointed by the Board of Regents and one by the Governor, who has designated Dr. George J. Fisher, a graduate in medicine of the Cincinnati College of Medicine and Surgery, 1898, editor of Physical Training, who has had large experience as a teacher of physical training and prominent in Boy Scout work.

---

**Training Camps for Medical Men** were conducted at Plattsburg, for 2-week periods beginning July 12 and 24.

---

**The General Education Board,** 61 Broadway, N. Y. City, has appropriated $2,920,874 for medical schools, out of a total of $13,386,968. This represents a far greater proportionate recognition of the needs and merits of medical education than is usually shown by either private or public philanthropies.

---

**National Board of Medical Examiners of the U. S.** This board was established largely through the interest of the late Dr. W. L. Rodman, President of the A. M. A. 1915, and is endorsed by the A. M. A. and a number of other national and regional societies. The provisional board consists of Surgeon General W. C. Braisted, U. S. N., President; the surgeons general of the other government services, a representative from the Federation of State Medical Examining Boards and a number of physicians prominent in educational lines. The permanent board is to include the Surgeons general of the three government services and one other member from each, three representatives of the Federation of State Medical Examining Boards and six members at large to be chosen by the board itself. Quarters have been assigned in the Army Medical Museum, for examinations and facilities for examination in laboratory work, etc., will be afforded by other government medical institutions. For medical graduates of 1912 and later, a 4-year high school course and 2 years of college work including physics, chemistry, biology and a modern language will be required; also graduation from a Class A college and one year's service as interne in an acceptable hospital or laboratory. Equivalent credentials may be accepted by the board for graduates prior to 1912. The examination will be rated according to the following schedule:

| | |
|---|---|
| 1. Anatomy | 100 |
| 2. Physiology | 75 |
| 3. Chemistry and Physics | 75 |
| 4. Pathology and Bacteriology | 100 |
| 5. Materia Medica, Pharmacology, and Therapeutics | 75 |
| 6. Medicine | 200 |
| 7. Surgery | 200 |
| 8. Obstetrics and Gynecology | 100 |
| 9. Hygiene and Sanitation | 50 |
| 10. Medical Jurisprudence | 25 |
| Total | 1000 |

A passing mark of 75% will be required in each subject. The fee for registration is $5. The certificate issued by the board has no present value toward obtaining a license but it is expected that it will ultimately be recognized by all state boards. A second but no further examinations will be allowed in case of failure. This is a great step in the right direction. It may be questioned whether educational requirements in advance of those established by law, for any given state and period should be made—at least without giving candidates the privilege of making up the requirements by private study. Still more questionable is the restriction to Class A colleges and "acceptable" hospitals. The selection of a medical school is usually made at an immature age and subject to various local and economic considerations. Appointment to an acceptable hospital is often entirely beyond the powers of the graduate and is not entirely dependent on individual merit. It is impossible from the mere numeric factors involved, that every graduate should obtain a satisfactory hospital position. Due allowance should be made for these limitations and, at least, opportunity should be given for making up deficiencies in early training of any kind. This is not merely the expression of an opinion based on democratic principles; we do not see how legal acceptance of the certificates issued by the Board can be accomplished unless full recognition is given to the legal status of all possible candidates in accordance with legislation existing at the time of their matriculation. Otherwise, while the certificate may be an honor, it is doubtful whether it can serve as a pass port to licensure.

---

**Actions in the U. S. Court** have been begun as follows: against Gioia, Bellanca & Co. with adulterating macaroni, shipped from Rochester to Scranton, by use of artificial coloring matter and inferior grade of flour; also labeling as made in Italy a product made in Rochester; against S. C. Wells & Co. of Leroy, with misbranding (in the sense of attributing therapeutic powers not established) a product called Dr. Carter's K. & B. Tea; against the Moone Chemical Co. (for the same reasons) for misbranding a product known as Emerald Oil.

---

**Shrine Convention in Buffalo, Week of July 10.** Dr. Edward A. Southall served as Chairman of the Committee on General Medical Safety and Dr. C. E. Stewart took special charge of the bands and patrols. Boy Scouts in large numbers and several city nurses, assigned by the Health Commissioner, assisted. About 80 cases, mainly heat prostration and minor accidents were treated, none being serious. The Homoeopathic Hospital donated its services to Shriners. In spite of the enormous congestion of traffic, especially on the evening of the parade when streams were diverted onto streets usually little crowded, and without available traffic police, no serious auto accidents were reported.

---

**The Vinum Cardui Suit** against the A. M. A. has been won by the plaintiffs, one cent damages having been awarded. Of course, court costs, attorneys' fees and even the publication of the full notes of the trial, have totaled a sum that would be staggering to a private enterprise. We can understand the nominal damage verdict in cases of innocent and harmless trespass on property or of assault under extenuating circumstances, where the general principles of property and personal rights are well established and must be upheld but when no real injury has been committed or the offender appeals to a sense of mercy by his weakness or is justified by general ethical principles contrary to the letter of the law. But, in the present case, a real damage has undoubtedly been done to the plaintiff. With no excitement or personal malice but for reasons which the medical profession at least can understand, there was the deliberate intent to inflict such damage. The A. M. A. makes no appeal for clemency on the ground of weakness. It is a strong body, influentially, numerically and financially. While the damages originally asked, $300,000, may be considered excessive and would have been a serious loss to the A. M. A., no ordinary amount of damages would have materially increased the expense incurred by the suit. It is true that the medical profession is almost united in believing that the ethical principles involved surpass the legal principles involved in published libels but the case has no analogy to violations of the law when personal honor or deep emotions are called into account. The case is one of many that might potentially arise and involves a general principle. Thus we feel that it would have been more satisfactory even to the defendant if the decision had been more definite. Either an organization considered as such, or a publication, has the right to denounce the claims and methods of those whom it regards, in good faith, as wrong doers, or professional considerations of ethics must be subordinated to the law as it stands. The future policy of the medical profession—and by analogy, other professions and organizations in what each regards as reform propaganda —would have been definitely established by a dismissal of the claim. If substantial damages had been awarded, it would have determined either a policy of very mild protest or would have opened the way to seek legislation which would allow reform propaganda of a vigorous nature. Under present conditions, no one knows where the matter stands. Each side has suffered a financial loss and neither can claim a substantial victory.

---

**Death of Centenarian.** Mrs. Mary Monroe of Binghamton, died June 28, after a brief illness, aged 105 years and 8 months.

---

**Chlorin Treatment of Water,** is being considered by Geneseo, which is supplied from Conesus Lake. It is also proposed to cover the reservoir to prevent the growth of red algae which produce a characteristic fishy taste. We understand that no gross contamination has occurred, although there is always the danger of typhoid when the water shed cannot be completely guarded.

**The Metric System.** The European War is undoubtedly familiarizing our people with metric units. One news report states that the German government is seizing considerable quantities of rubber, supposed to be smuggled into the country, paying for at the rate of 5 marks per "goligram."

---

**Sunstroke.** As noted, the Shriners' parade in Buffalo, although engaging large numbers, including many elderly men and having the unfavorable factors of a considerable exposure to the hot sun in apparently heavy uniforms, resulted in only a few mild cases of heat prostration. Two or three spectators were similarly affected. A moderate number of other cases have been reported. The first death of the season occurred in Buffalo, July 12, in the person of a laborer, aged 50, in the Erie R. R. yards. Rochester had one death the same day, the temperature being 92 and the humidity excessive. Four deaths from sunstroke occurred in Buffalo on July 20, and five other serious cases were under observation.

---

**Niagara County Tuberculosis Hospital.** The Joint Committee has approved the site south of the old almshouse farm, near Lockport, and the Lockport Board of Commerce, Niagara Falls Board of Trade and Niagara Falls Tuberculosis committee have added their endorsements. $100,000 has been appropriated for a hospital, originally planned to have 65-75 beds but the Joint Committee estimates that 100 persons actually needed such accommodations at present.

---

**Infantile Paralysis Epidemic.** 277 cases were reported in N. Y. City for June and 10 outside the city but mostly in adjacent counties. Up to July 11, 1278 cases had been reported in N. Y. City with 270 deaths; 26 cases outside the city for June and 48 since July 1. (Note: The discrepancy in the up-state figures is undoubtedly due to delays in diagnosis and reports). July 15, the N. Y. City cases reported for the 24 hours ending 10 A. M. numbered 144 with 27 deaths. July 16, new cases numbered 96, deaths 17. The first Buffalo case developed July 14, in a child who had visited in Brooklyn. A second case was reported July 17 but the diagnosis was later changed. July 19, the N. Y. City cases reported in the last 24 hours numbered 143 and the deaths 30. Dr. Chas. E. Banks of the U. S. Public Health Service assumed charge of the fight against the epidemic at this date. July 19, the up-state reports for the last 24 hours were 15 with no deaths, the total of up-state cases to date being 161. We have received no confirmation of the report that the germ has been isolated. The campaign is being waged by both Federal and State and local health authorities in accordance with principles familiar to the profession and every effort is being made toward public instruction. July 20 N. Y. City cases totaled 2246, deaths 458. July 21 a case was reported from Warsaw, and two doubtful ones from Dunkirk. July 23 one of the Dunkirk cases was reported positively, the total up-state cases being 211. July 22 the N. Y. City cases reported for the 24 hours, numbered 135 with 39 deaths; July 23, 115 cases with 23 deaths. The State Board of Health has made plans for the establishment of temporary laboratories, two on Long Island, as Brooklyn is regarded as the most serious center of infection, and one near Kingston. Adrenalin has been announced as favorably influencing the course of the disease.

---

**Care for Superannuated Physicians.** We earnestly request our readers to reply to the questions at the end of the editorial in the July issue. Some have been received already but the matter can reach a practical issue only by co-operation.

---

**A Professional By-Product.** The Journal of the Indiana State Medical Assn. repeats, as a definite occurrence, the old story of the man who engaged a physician to attend his wife, at some distance, rode out with him—this time in an auto, the story first appearing prior to the horseless age—discharged and paid him on his arrival, explaining that the charge was less than for a livery. Some years ago, a country physician offered an almost illiterate farmer half of his professional earnings, if the latter would furnish him a "rig" and drive him to his calls; and the offer was refused. The average professional fee would not, as a rule, exceed the charge for transportation to and from the patient's house, by a horse or auto livery. Neither is it more than a plumber would charge, say for thawing out a pipe that was not burst. It is somewhat humiliating to have thus impressed the economic fact that the average professional service is about equal to that of a comparatively uneducated and unskilled laborer, plus a horse and buggy or automobile or set of tools. Still, there is something to be said on the other side. All of us are guilty, if the term be allowable, of purchasing a service or a commodity for its by-product. We take a Turkish bath as a substitute for a hotel room, or a street car trip to keep dry in a sudden shower, or we buy an article or subscribe to a magazine for the sake of the premium. If a physician can afford to make a professional call and furnish his transportation for a price lower than that of the average livery—as he certainly can and usually does—one may view philosophically the occasional times when we are used for the latter purpose, alone. It happens to most of us once in a life time. If our services compare in actual demand-value, with some very humble vocations, it is only fair to remember that, theoretically and to a large degree practically, we represent about the same kind of human material as enters into these other industries. Moreover, like any other industry, medicine is paid according to the law of supply and demand and no one can question but that, with some few local exceptions, the supply very much exceeds the demand. Again, education is a wide-spread, easily available factor in human value. Very seldom does it involve serious hardship in these days. On the contrary, it is a rather pleasant occupation. For work of the ordinary kind, manual or clerical, it makes little practical difference to the purchaser of labor or its products, whether the labor emanates from one who has had the minimum which the government now allows or the few years' more which the government urges him to accept free, except for his maintenance. Thus, generally speaking, we do not find any conspicuous difference between the pay of the grammar school and the high school graduate, after a few years. With about a tenth of the youth of the country possessed of a high school education and with a large proportion of young adults continuing their education by attendance at night schools, by reading and use of lecture courses, museums, etc., and by definite training along some particular line of usefulness, the devotion of a few years more of adolescent life to formal educational, scholastic or professional or both, does not create an abyss between ordinary trades and businesses and the so-called learned professions, which purchasers of the ultimate products of human industry will regard with much awe. In one sense, education remains a priceless jewel but we cannot place it within the reach of all who will make a little effort or who have a normal parental support and at the same time, maintain its former high commercial value. If we rate our professional dignity in financial terms, we must not blame the public for basing our financial value on a commercial basis of what our services are worth according to market conditions, especially when we ourselves are to blame for overstocking the market.

